# Epidemiology of Plague: Problems with the Use of Mathematical Epidemiological Models in Plague Research and the Question of Transmission by Human Fleas and Lice

**DOI:** 10.1155/2019/1542024

**Published:** 2019-08-18

**Authors:** Ole J. Benedictow

**Affiliations:** University of Oslo, Department of Archaeology, Conservation and History, Section of History, Oslo, Norway

## Abstract

This article addresses the recent use of mathematical epidemiological SIR or SEIR models in plague research. This use of S(E)IR models is highly problematic, but the problems are not presented and considered. Serious problems show in that such models are used to “prove” that historical plague was a (1) Filoviridae disease and (2) a bacterial disease caused by *Yersinia pestis* which was transmitted by human fleas and lice. (3) They also support early-phase transmission (by fleas). They purportedly consistently disprove (4) the conventional view that plague is/was a rat-and-rat-flea-borne disease. For these reasons, the focus is on methodological problems and on empirical testing by modern medical, entomological, and historical epidemiological data. An important or predominant vectorial role in plague epidemics for human fleas and lice requires that several necessary conditions are satisfied, which are generally not considered by advocates of the human ectoparasite hypothesis of plague transmission: (1) the prevalence and levels of human plague bacteraemia (human plague cases as sources of infection of feeding human ectoparasites); (2) the general size of blood meals ingested by human fleas and lice; (3) the consequent number of ingested plague bacteria; (4) the lethal dose of bacteria for 50% of a normal sample of infected human beings, LD_50_; and (5) efficient mechanism of transmission by lice and by fleas. The factual answers to these crucial questions can be ascertained and shown to invalidate the human ectoparasite hypothesis. The view of the standard works on plague has been corroborated, that bubonic plague, historical and modern, is/was a rat-and-rat-flea-borne disease caused by *Yersinia pestis*. These conclusions are concordant with and corroborate recent studies which, by laboratory experiments, invalidated the early-transmission hypothesis as a mechanism of transmission of LDs to humans in plague epidemics and removed this solution to the problem of transmission by human fleas.

## 1. Introduction

This article does not criticize the S(E)IR models or other mathematical epidemiological models per se but the erroneous use of them, in this case on historical plague epidemics. The S(E)IR models are good for their intended use and do not need further development, if possible. What is needed is a higher awareness and better understanding of their area of application, the range and limitations of uses, and better historically relevant scholarly competence.

It seems to have become a usual misconception that models can be used to prove or corroborate views or assertions on some aspect of reality, to produce empirically valid inferences (at some level of tenability). This has clearly been the case in historical plague research with significance also for the understanding of modern bubonic plague. However, models are in methodological principle analogues and can, as such, only be used to engender or develop (working) hypotheses by seeming similarity. Epidemiology can be considered a medical branch of sociology relating to the spread of disease by human behaviour in interaction with diverse social contexts. The view on models formulated in, e.g., *Modern Dictionary of* Sociology, is useful:

model: A pattern of relationships, either conceptual or mathematical, which is found in some way to imitate, duplicate, or analogously illustrate a pattern of relationships in one's observations of the world, such as patterns in social behaviour or social structure [.] No single model or combination of models reveals the truth of the structure of reality. Each model is determined by its usefulness for guiding study.

And they add under the entry of “model, mathematical”:

*Many sociologists tend to feel that mathematical models, which have been useful in the physical sciences, are not meaningfully applicable to the logic and patterns of social life* [1].

The use of the verb “feel” is unfortunate and should be replaced by “think” or “consider” rather. Also epidemiological mathematical models are inherently analogues, based on an analogy between the spread of contagion in a human population according to “the logic and patterns of social life.” In the case of plague, this premise also includes the “logic and patterns” of behavioural strategies of specific species of fleas and (black) rats and purportedly but erroneously also of human lice (see below). This also shows in the term simulation modelling where models are manipulated by various data until a satisfactory and often preconceived outcome is achieved.

According to the methodology of (social) science, analogues cannot be true (or false) or prove something to be true (at some level of tenability). The essence of models is that they can provide guidance for the construction of working hypotheses that can be empirically adequately tested or of experiments which provide empirical data suitable for testing. Scientists tend to ignore that many social scientists consider that mathematical models are not “meaningfully applicable” in the study of social contexts and, in casu of historical (plague) epidemics. Historians consider that the study of historical epidemics requires good knowledge and understanding of the specificity of the historical society in question: the functions of the interaction of its economic, political-administrative, and cultural structures as expressed in people's activities, beliefs, behavioural patterns, and motives, which structured the societies and engendered the dynamics of epidemic spread.

Relating to the theory of early-phase transmission of plague by fleas, B. J. Hinnebusch (Chief of the Plague Section of the Rocky Mountain Laboratories, NIH, NIAID) pointed out, among other things, that results presented by scientists on the purported efficiency of early-phase transmission apparently were based on mathematical models using estimates of important parameters with little or no experimental (i.e., empirical) support [2]. Recently, the validity of this serious criticism was demonstrated when he showed, together with two coauthors, that the claims associated with the original experiments could not be reproduced. The results of the mathematical model were ipso facto shown to be untenable and that early-phase transmission would be significant only in highly susceptible rodent populations experiencing a high flea burden and not in the case of human plague [3, 4].

Epidemiological mathematical models produce the outcome of the selection of data fed into them and can support the claims researchers find useful. This will be shown also below in the presentation of studies where scientists using the same mathematical model, the so-called Reed–Frost (SIR) mathematical epidemiological model, claim to prove the validity of two entirely different and incompatible alternative theories on the microbiological identity and epidemiological dynamics of the Black Death and historical plague epidemics.

## 2. Basic Structures of the Reed–Frost Mathematical Epidemiological Model and Other SIR Models

The Reed–Frost mathematical epidemiological model is central, and it has been used by Scott and Duncan [5], Christakos et al. [6] (and in several other articles below), and, in reality, by K.R. Dean [7], Whittles and X. Didelot [8], and Dean et al. [9], purportedly to prove alternative theories on the microbiological nature and/or the epidemiology of the Black Death and the subsequent epidemics of the second plague pandemic. The model's name refers to two scholars at the Johns Hopkins School of Medicine, L. J. Reed and W. H. Frost who worked on it in the late 1920s.

The Reed–Frost model is a variant of the SIR model for spread of disease. Taking into consideration several highly restrictive conditions, this type of model can be assumed to be plausibly predictive for the epidemiological development of a number of viral infectious diseases which are transmitted by direct cross infection by physical contact or via infected droplets and which also confer lasting immunity, such as measles, flu, mumps, rubella, and chicken pox [10].

Bacterial diseases are not included, also because they quite generally do not confer lasting immunity on survivors. This is also the case with bubonic plague, and survivors generally acquire only weak and brief immunity [11]. This is reflected in the numerous cases of survivors of plague disease who fall ill by plague again and also three times in the same epidemic who are mentioned in historical sources [12]. This was also noted in connection with modern bubonic plague in India, China, and Vietnam, [13, 14]; Langen and Liechtenstein state on the experience with plague epidemics in Java: “reinfection with plague may occur within a few months; surviving an attack gives but a very limited immunity” [15]. This shows, for starters, that bubonic plague cannot be modelled by SIR models including the Reed–Frost version of the model because it is a bacterial disease and because it does not confer lasting immunity.

The so-called Reed–Frost model was not published by Reed and Frost, and they never called it the Reed–Frost model. It was first scholarly presented by J. De Oliveira Costa Maia in the *Journal of Human Biology* in 1952 with strong emphasis on the model's great limitations and shortcomings. Maia emphasized on the first page that “As presented, the theory applies only to simple situations as illustrated by an outbreak of measles in a closed group” [16], and the latter is another general condition for the use of a SIR model. A closed group is a theoretical demographic or epidemiological concept referring to a constant or a self-contained group/community of people without emigration or immigration of members (including births), an artificial social construction which offers certain opportunities for hypothetical demographic and epidemiological analyses. It is accompanied by other artificial conditions inherent in the SIR model spelled out by Maia: “[.] a closed population [.] in which people intermingle fairly uniformly,” because that makes it “plausible” that “every individual will have the same number of contacts.”

Maia also underscored that the underlying theory of the model “cannot explain,” for instance, “Diseases with multiple hosts, such as insect vectors and animal reservoirs” [16], which also are general conditions for the use of SIR-based models. This means that Maia made it entirely clear that the Reed–Frost SIR model could not be applied on epidemics that were or were suspected of being bubonic plague, which are by wide consensus transmitted and disseminated by bloodsucking (hematophagous) insects and associated with rats and rodent reservoirs. Such use would independently, on both the latter points, be fallacious. By implication, this applies generally to SIR-based models.

The editors of *Human Biology* entertained so many lingering reservations, as it seems, that they let Maia's article be immediately followed by an article by H. Abbey of the Department of Biostatistics at the Johns Hopkins School of Medicine, a later colleague of Reed and Frost. In this article, she devastatingly demonstrated its serious weaknesses and why it never had been published. The Reed–Frost model was only poorly usable for the study of the spread of viral diseases in closed populations and had been used pedagogically in the teaching of medical students at the School [17].

Abbey added some specific structures of the closed group that all members had equal susceptibility to the disease in question, equal capacity to transmit it, and the power of passing out of observation when the transmitting period was over, which supplements the number of basic conditions of the SIR mathematical epidemiological model and demonstrates their highly restricted, artificial, and hypothetical functionality. The Reed–Frost version of the model was not published or used because other well-established mathematical epidemiological models functioned much better. This explains that the so-called Reed–Frost theory long remained practically unknown.

Summing up, also the Reed–Frost variant of a SIR model can only be applied to the study of viral infections which confer lasting immunity in survivors, are spread by cross infection by direct physical contact or by droplets [17] in a closed group where people intermingle uniformly and have the same number of contacts and exposure to infection. Conversely, for a number of necessary conditions with separate powers of invalidation, it cannot be applied on epidemic diseases that (1) are or are suspected of being bacterial, (2) do not confer lasting immunity, (3) are conferred by intermediary agents, such as insects, and/or (4) have animal reservoirs, as in the case of bubonic plague. Furthermore, (5) people in historical society did not, by any stretch of sociological imagination, intermingle according to a pattern providing a uniform level of exposure to infection. The social scenes of historical plague epidemics were never “simple situations” as all historians know.

## 3. The Use of the Reed–Frost SIR Model to Prove That Historical Plague Was a Filoviridae Disease

### 3.1. Scott and Duncan's Use of the Reed–Frost Model in Historical Plague Research

The Reed–Frost mathematical epidemiological model was first presented and used by Scott and Duncan in their 2001 book to argue for an entirely new theory of the microbiological nature and epidemiology of historical plague. They refer only to Maia's article as the source of their information on the model [5]. They did not refer to Abbeys sharply critical article or another later critical article [18].

Scott and Duncan used the Reed–Frost model to analyse various historical plague epidemics, and it consistently confirmed the revolutionary theory that historical plague had to be a Filoviridae disease, (a variant of) Ebola disease, or Marburg disease [5]. According to the Reed–Frost model, historical plague epidemics allegedly exhibited a pattern of spread formed by the direct exchange of infected cells containing viral particles between a diseased [5] (or objects contaminated by fresh body fluid of a diseased) and a healthy person [19–21]. This type of disease has never been transmitted outside a range of African countries stretching from the southern coasts of West Africa to Sudan, where it has a zootic reservoir [22]. In this zootic reservoir, Filoviridae diseases circulate among animals which do not live in the territories ravaged by the Black Death and the second pandemic or in the countries where the third plague pandemic spread. Scott and Duncan performed these analyses without discussing the methodological principles or problems involved in the use of the Reed–Frost theory and without discussing them in the light of the array of necessary conditions for its use laid out by Maia (and Abbey). In this context, let it suffice to point out that (1) epidemics of bubonic plague in general do not conform to the pattern of spread of Filoviridae epidemics formed by direct physical transmission of viral particles in cells, as observed in Africa [11, 23–28], and (2) that scientific studies on Filoviridae diseases do not contain a single human case with bubo(es), a defining feature of modern and historical plague [11] (although rare cases of enlarged lymph nodes have been reported, as should be expected) [29].

In a treatise designed to show that historical plague cannot have been bubonic plague but was a Filoviridae disease, Scott and Duncan tacitly chose to use an epidemiological model which cannot be used to test historical evidence on epidemics which were or could be suspected of being due to bacterial infection or transmitted by insects, such as bubonic plague. As such the whole treatise is based on a fallacy of methodology. This should have been known by Scott and Duncan who explicitly inform that they base their knowledge of the Reed–Frost model on Maia's article. Indirectly, this is corroborated by a statement on p. 355: “typhus epidemics do not follow Reed and Frost dynamics because it is a disease with an arthropod vector.”

## 4. Christakos Et Al.'s Use of the Reed–Frost Model in Historical Plague Research

### 4.1. The Selection of Model and Disease

In 2005, Christakos et al. published a treatise focused on epidemic modelling of the Black Death. It was likewise based on the application of the Reed–Frost model with explicit references to Scott and Duncan's use of it and as originally presented by Maia [6]. They do so despite having noted, as it transpires from a paper published about simultaneously, that Scott and Duncan emphasized that the Reed–Frost model “can only explain simple infectious diseases and cannot represent infections with multiple hosts, such as the bubonic plague” [30]. They also do not specify that these restrictions also included bacterial diseases, such as plague. The fact that the Reed–Frost model cannot be used to “represent,” i.e., to model bubonic plague for several sufficient conditions, because it is a bacterial disease with an animal reservoir and transmitted by insects, means in methodological terms that that the Reed–Frost model cannot be used when a central issue is whether or not historical plague was bubonic plague, a crucial condition also ignored by Scott and Duncan.

Christakos et al. purportedly confirmed Scott and Duncan's theory that the Black Death was a Filoviridae disease. They also refer to “the good agreement between fatalities predicted by the extended Reed–Frost (ERF) model and the actual mortality data” [6]. The same year, Olea and Christakos, and vice versa, published two collateral articles on the Black Death, one on the duration of urban epidemics and another on the space-time characteristics, based on selections of the same material and to the same effect [30, 31].

None of the five authors are medievalists, in the eyes of a medievalist and a medievalist demographer, this shows. To give a representative example, they state that the only piece of data on the mortality in the Black Death in Scandinavia is a mortality rate of 50% in Oslo, which they had found on the Internet, [6] more precisely at htttps://www.lonelyplanet.com [32], a source of this information that I have not succeeded in tracking down. In view of the fact that there is not any documentary evidence about the pre-plague size of Oslo's population or on the mortality in the Black Death or on the size of Oslo's population for a long time to come, it is evidently a fantasy figure. On the same page, they refer to my 1992 doctoral thesis on the Black Death and subsequent late-medieval plague epidemics in the Scandinavian countries (and to my 2004 monograph on the Black Death) where this is made clear [28, 33].

As for their selection and use of the Reed–Frost theory, they mention Maia's article twice as a bare reference of recognition without material content [6]. They display no specific independent knowledge of Maia's article and do not mention the strong limitations and special conditions for valid use that he presented.

Instead, they present it incorrectly in general terms as “the well-known model” of Reed and Frost but only mention Maia (1952) and Scott and Duncan (2001). This implies that their source of information on the Reed–Frost SIR model is second-hand, taken from the latter work where it has been selected and formed for the two authors' use, which explains that they do not consider any of the strong reservations and limitations emphasized by Maia. This can also explain that Abbey's article [17] is unknown to all five authors. They mention that there “is no shortage of epidemic models,” but claim that all of them, “despite their sophistication,” are more deficient than the Reed–Frost model because they “seem to disregard” that “in real-world situations, the epidemic variables are fundamentally spatiotemporal” [6]. This is evidently not correct. Moreover, here the term spatiotemporal is used as an empty concept. A historian or social scientist would have used or added a term implying a specific societal texture with specific properties for dissemination of epidemic disease. In practice, Christakos et al. use temporal models without spatial variables. Clearly, the general presentation of the Reed–Frost model and the argument for the choice of this model are sharply at variance with Maia's (and Abbey's) views. There is no indication that the Reed–Frost SIR model should be a well-known, highly considered, or much used model.

Christakos et al. supplemented the Reed–Frost model with the so-called Bayesian maximum entropy (BME) mathematical method. The inclusion of BME purportedly makes it possible to produce space-time epidemic maps that “account for the spatiotemporal characteristics of the Black Death” in the form of “correlations between spatial and temporal mortality structures” and much more. This is supposed to introduce the spatial dimension of the development of epidemic mortality which allows identification of epidemic diseases according to characteristic spatiotemporal features, in this case implicitly irrespective of the kind of contagion or means of transmission and spread [6, 30, 34]. The space-time BME maps of the spread of the Black Death presented later are clearly false and incompatible with the historical sources as presented in my 2004 book on the Black Death [6].

In this book, the spread of the Black Death in the years 1346–53 is presented over 180 pages on the basis of all available historical evidence at the time, handled according to the medievalist historian's craft, and in corresponding empirical detail. This maximum empirical material is analysed, epidemiologically structured, and put in a superordinate or synthetical epidemiological perspective in a separate section called “Patterns and Dynamics of the Black Death.” On this basis, it was concluded that all movements and features of the spread of the Black Death conformed to the conventional view of bubonic plague as rat-and-rat-flea-borne [28], as presented in all standard works on this disease [11, 14, 23–26]. They do not dispute or challenge this material or the synthesis but tacitly pass them by.

The unusefulness also of BME mathematical models shows already in the next chapter where spatiality in reality is absent as an epidemic factor or dimension in their material, also in the form of population density. It is demonstrated also by the fact that the purportedly identified (Filoviridae) disease has been shown to be completely erroneous (see below).

## 5. The Generalized Concept of City: How to Avoid the Inverse Correlation between Population Density and Plague Mortality as a Defining Feature of the Black Death

For the central thrust of their study, Christakos et al. claim to have established and analysed “urban data from 53 cities, with 200 to 120,000 residents.” The defining numbers show that their data include settlements of all sizes from small village (or hamlet) to metropolis [6, 26]. The generalized usage of the words urban and city to signify the whole range of sizes of human settlements is unexplained, unprecedented, and at variance with common usage, and with the usage of historians and social scientists, and also with the definitions of dictionaries of English, which are similar. According to the *Cambridge Dictionary online*, “A city is a large town,” a town is “usually larger than a village but smaller than a city,” a village is “a group of houses and other buildings that is smaller than a town, usually in the countryside,” and a metropolis is “a very large city.” According to the same dictionary, the concept of urban has the meaning “of or in a city or town” [35]. Also Christakos et al.'s usage of the concept of urban is, unexplained and unprecedented, also used synonymously with city to cover also the full range of rural settlements, from small village (or hamlet) to large village. In Table 8 of their book, for instance, the English manors of Cuxham and Fingrith are designated cities together with big cities, such as Bruges and Rouen, and together with metropolises such as Paris and Florence [6, 31].

This does not mean that the five authors do not know the meaning of the word city and its conceptual relationships with other concepts denoting various categories of settlement size with associated social and economic structures. It is the strained solution of choice to an insurmountable problem. In the specific context, this usage of the concept of city contains a central assertion that demographic structures of population size and density combined with various social and economic structures do not significantly affect epidemiological functionality: this include the exposure to infection and the susceptibility to importation of diseases into local society, the powers of spread of epidemic diseases within settlements of all sizes irrespective of economic and social structures. Whatever combination of these demographic, social, and economic categories, they allegedly produce similar rates of morbidity and mortality. Christakos et al. stated that they wished to include spatial variables, but instead appear to make efforts to exclude spatial variables, in this case associated with the crucial epidemiological dimensions of population size and density.

In fact, Christakos et al. assert repeatedly that, according to evidence produced in India, modern bubonic plague was “a rural disease hitting harder in the small villages than in the countryside” (a strange formulation) and emphasize repeatedly, twice in the book and also in two of their collateral articles, as their crucial finding, that their study of the Black Death “shows a distinct absence of correlation between city size and mortality,” or “reveals no correlation between city size and mortality” [6, 30, 34]. One must here not be confused by their unprecedented use of the term “city size,” which refers to settlements of all sizes. They assert (1) that the Black Death produced the same level of mortality irrespective of population size and density in combination with any social or economic structures and (2) by implication that this is a proof that the Black Death was a different disease from the modern epidemics of bubonic plague in India.

This is at variance with the central tenets of epidemiology as formulated, e.g., by F. Macfarlane Burnet, the Nobel Laureate, in his standard work on infectious diseases:

*no matter by what method a parasite passes from host to host, an increased density of the susceptible population will facilitate its spread from infected to uninfected individuals* [36].

The powers of spread of all infectious diseases transmitted by cross infection between hosts increase with increasing density of susceptible population. This would unconditionally also be the case with Filoviridae diseases that are transmitted directly from diseased to healthy persons. Consequently, when Christakos et al. find, nonetheless, that this was not the case with the Black Death, it is due to the erroneous identification of the disease as a Filoviridae and the fallacious use of a mathematical epidemiological model.

This topic contains other important and surprising insights. For their Indian evidence, Christakos et al. consistently refer only to E. H. Hankin's 1905 article where this point is indeed made but within a much broader scope. It was made in the context of statistical material showing generally falling mortality rates with increasing size of settlements, and therefore, much higher mortality rates in small villages than in large villages where it was higher than in towns and cities. Hankin's material and argument are not confined to rural society but also include towns and cities. Christakos et al. refrain from mentioning that Hankin also compared his surprising finding with available English evidence on the Black Death and found evidence of a similar tendency. These empirically supported observations are conspicuously at variance with Christakos et al.'s consistent finding by use of an analogous mathematical epidemiological model that population size and density and social and economic structures were without epidemiological significance [37]. Hankin made an important pioneering observation of a unique and defining feature of bubonic plague that modern bubonic plague in India and the Black Death in Europe had in common, the inverse correlation between population density and mortality rates. Adequately presented, Hankin's article contains evidence that the Black Death had similar epidemiological properties as modern plague in India and was bubonic plague.

Hankin was not alone in making these peculiar observations. The Indian Plague Research Commission included an epidemiologist and statistician, M. Greenwood, who a few years later made another study of this topic. On a much broader statistical basis, Greenwood unconditionally confirmed Hankin's finding. Also he consulted the available evidence on the Black Death in England and found a similar corroboration: bubonic plague in India in the early 1900s and the Black Death shared this distinguishing feature that mortality rates were inversely correlated with population size and density along the spectrum from small villages to towns and cities, and this was a unique and defining feature of bubonic plague [38].

In their book and collateral articles, G. Christakos and A. Olea with collaborators refer repeatedly to my 2004 book on the Black Death. In this book, this defining feature of bubonic plague is presented in a chapter containing both modern and historical evidence [28]. It was based on my previous in-depth study in a long article on the topic [39]. It is also stated that it would be important to look for similar evidence in this complete study of the mortality of the Black Death, and indeed, supporting evidence of the inverse correlation between mortality and population size was found and no evidence to the contrary. This confirmed the quality also of Hankin's and Greenwood's associated comparative historical studies and perspectives [12, 28].

Hankin and Greenwood were rightly convinced of the validity of their finding but could not explain it. In a sweeping and general fashion, the central principle of epidemiology predicts that no matter by what means or method a disease is transmitted from person to person, morbidity and mortality rates will increase with increasing density of the susceptible population. However, diseases that are not transmitted directly from person to person, such as, for instance, rat-and-rat-flea-based bubonic plague, can exhibit a different pattern. In the case of diseases spread by interhuman cross infection, the density pattern is, so to speak, one-dimensional, comprising only humans: the density of susceptible humans will decide the powers of spread. In the case of rat-and-rat-flea-based plague, the density pattern is three-dimensional, comprising the density not only of humans but also of rats and rat fleas. The latter two density factors will tend to covary strongly and, therefore, to override the significance of the density of humans. Rats are social animals defending territories. This means that, in the countryside, at least one rat colony will normally coreside with a household, among the substantial tenantry other colonies often also in the barn, byre, or storerooms, whereas in urban environments several households would usually crowd together within the territory of a rat colony. The ratio of humans to rats and fleas will, accordingly, tend to be lower in urban environments than in rural settlements, and there would be more persons to share between them the dangerous rat fleas let loose from an afflicted rat colony [39].

This epidemiological model provides a basic explanation for why, in the case of bubonic plague, the severity of impact on human populations declines with mounting density of human settlement. This finding is of crucial importance. Only an epidemic disease with these disseminative properties could possibly have brought about the demographic developments observed in the Late Middle Ages when around 90% of the population lived in the countryside. Christakos et al.'s focus on urban plague is misplaced.

One point remains to be explained: the tendency of mortality rates to rise again in big cities and metropolises. Because they would be surrounded by walls, the price of building sites was so high that it induced the building of multistorey houses, quite often with two or more living units at the same level. This would create new ecological niches or habitats for black rats where colonies would settle in the space provided by floors, in the walls between living units, and in the ceiling or roof, according to a pattern observed also in India, “the fact that *R. rattus* lived at all levels of the houses and therefore in close contact with man” [40]. This would produce the effect of increasing the number of rats and fleas relative to the human population within the territories of rat colonies, causing mortality rates to rise again and to surpass the level characteristic of towns but not of villages.

Clearly, also this explanatory model of the powers of epidemic spread is density dependent but according to a three-dimensional density structure which functions differently from diseases with a monodimensional structure. Because this explanatory theory explains an empirically strongly underpinned unique pattern of mortality according to settlement size for bubonic plague, namely, the inverse correlation according to population density, it also constitutes evidence that modern and historical plague at least predominantly were the same disease, namely, rat-and-rat-flea-borne bubonic plague.

On p. 206, in Chapter V(D, f) on “Some Comparisons with Bubonic Plague,” Christakos et al. presented Figure 7, a map allegedly showing the main features of the “Propagation of the modern bubonic plague in India (adapted from Plague Research Commission, 1912).” In References A, the entry of this reference is supplemented with the page reference 207–242. This is apparently a spurious reference because no such map is presented by the IPRC anywhere in their reports: the given pages in volume 12 of 1912 contain IPRC's four reports (term here used for research articles) nos. XLVIII-L and half of report LI, which do not relate to the spread of plague in India. This is not a misprint or scribe's error, the volume of 1913 does not contain reports by the IPRC, and the volume of 1911 contains reports by IPRC's epidemiological specialist Greenwood to the opposite effect. As pointed out earlier, Hankin and Greenwood compared their studies of the spread of plague in India with the spread of the Black Death in England and concluded that the pattern of spread of these plague epidemics was similar. This apparently spurious map and reference with accompanying comments are the consequence of a completely erroneous identification of the disease and the distortions of the mathematical epidemiological model which engender a strong need for construed support.

All assertions to the effect that plague in India or China spread according to a different pattern from the Black Death, for instance, with respect to pace, except for the significance of spread by steamships and railways, are not based on epidemiological studies by IPRC [12] or on the Chinese plague research team based in Shanghai [41] or on the standard works on plague synthesizing (also) this plague research [11, 12, 23–25].

### 5.1. The Burial Register of Givry and the Latency Period of Plague Epidemics

Another defining feature of modern and historical bubonic plague formed by the rat-and-rat-flea-borne epidemiology can be demonstrated also by addressing another example of their model-based use of a historical source. This is the only extant continuous parish burial register and covers the Black Death in the town of Givry in Burgundy in France. The register is unpublished, and the basic data are known in the form of a table showing burials per day in the period of the Black Death [42]. Importantly, Christakos et al. made “a central decision,” namely, “to time mortality *every month*,” which required that they extended the temporal structure of the original *Reed–Frost* model as presented by Maia [6]. This shows that Christakos et al. did not choose to use the Reed–Frost model because it allegedly, in contrast to other epidemiological mathematical models, included not only temporal but also spatial variables. In practice, they use a mathematical epidemiological model with only a temporal variable and decided to extend the basic unit of temporal organization of the data, in this case the mortality data of the burial register. Although not explained, the temporal extension serves a purpose. As a consequence of the extension, all data displaying distinguishing epidemiological features with a shorter development period than the basic unit of temporal organization of a month will be blurred or disappear when they are fused with many other data.

Christakos et al. claim that the pattern of mortality from the first cases in early July 1349 to the last in mid-November can be shown by the Reed–Frost model to exhibit the clear characteristics of a Filoviridae disease. However, when studied according to the temporal unit of day, the early phases of the epidemic developments as displayed in the original table is, for all practical purposes, incompatible with Filoviridae diseases. Filoviridae diseases have a normal incubation period of 8–10 days (an exceptional case with an incubation period of 21 days is recorded), [43] when the course of illness with contagiousness begins with a median duration of 8 days (an interquartile range of 7 to 11) [44]. This is the case also for other viral diseases with relatively short courses of contagious illness which, by their epidemiological nature, are characterized by continuity of infection and cases. Instead, it is concordant with another defining feature of bubonic plague, the latency period, the “silent” period between the first arrival of an infective rat flea in a community to the first or next human death(s): on 5 July 1348, there were two burials corresponding to the normal (pre-plague) monthly mean, then followed a burial on 17 July and one the next day, which were followed by several brief breaks of a few days distributed among a day with a burial or a few days with a few burials, before the burials began to assume continuity by day in early August.

In the case of bubonic plague, the first deaths would reflect the arrival of one or more infective rat fleas by merchandise or with a traveller about ten days earlier. This estimate relates to a retrogressive timeline consisting of a couple of days from death to burial and eight days representing the average duration of the equally long normal courses of illness and time of incubation, indicating 25 June as the day plague contagion arrived. If this infective flea or another flea with the same provenance infected a preferred host, a rat, about the same day and triggered the beginning of the rat epizootic, a latency period of 19–27 days would ensue before the first human death occurred in this chain of epidemic events. This includes the duration of the rat epizootic of 10–14 days, 3 days of fasting for rat fleas that do not find a new rat host, and then leap onto human beings in their proximity, and the two periods of incubation and course of illness before the first human death [12, 45]. The average duration of this process indicates that the first human death(s) should occur on the 23rd ensuing day or exactly on 17 July. This long silent period, denoted a latency period, is a characteristic feature and defining feature of rat-based-rat-flea-borne bubonic plague [12].

In this case, the latency period is followed by the slow and “sputtering” development of the endemic phase of bubonic plague, reflecting the early epizootic phase in the rat colonies before it slowly translates into the early epidemic phase in early August. This shows that the usage of feeding mathematical models with burials according to units of time of a month veils or conceals important epidemiological features with defining properties for identification of the disease. Christakos et al. do not examine any other option than the Filoviridae theory, for instance, whether the form of the epidemic could conform to bubonic plague, which could not be performed by model, only by epidemic analysis.

There is much more epidemiological evidence supporting the notion that the disease was ordinary bubonic plague. The slowly developing early epidemic phase in August with a few burials about every day was followed by a sudden strong upsurge in burials in September which reflects the widespread and intense phase of the underlying rat epizootic. At the turn of the month, a slow decline set in followed by a strong decline from mid-October and the disappearance of burials in the third week of November. In the spring, the Black Death broke out in this region and expanded widely [12, 28, 46]. Christakos et al. do not attempt to explain why a disease spread by direct transmission of contaminated living cells between diseased and healthy persons should display this rhythm of seasonal development: the association with warm and temperate weather, the disappearance with cold and wintry weather, and why or how it should recrudesce after a long winter break. In winters, people crowded closer together to keep warm and reduce fuel expenses, which reduces interpersonal distance and enhances infection by contact, droplets, and lice. Without alternative empirical-based explanation, this usual epidemic pattern of bubonic plague invalidates their model-based hypothesis (see below).

The inverse correlation between mortality and population density and the latency period are two defining features of rat-and-rat-flea-borne bubonic plague that can be identified by the individualized study of number of deaths by day. Otherwise, there is no functional alternative to the usual work-intensive practice of historical demographers and historians, to follow the spread from person to person, from habitation to habitation, within families and between families, from street to street, such as performed, for instance, by P. Slack and J. G. Dijkstra, which reveals the specific pattern of rat-and-rat-flea-borne bubonic plague [47, 48].

### 5.2. Clinical Features as Evidence

The Filoviridae theory can also be tested on clinical aspects. As pointed out earlier, no human case of Filoviridae disease has presented with a bubo, also the protagonists of this alternative theory do not refer to any known case. In contrast, human cases of bubonic plague normally exhibit buboes both in modern and historical plague, most often in corporal areas with concentrations of lymph nodes, in or near the groin, in the armpits, and on the neck. This reflects that infective fleas deposit contagion at an intradermal level in the catchment area of the lymphatic system. Buboes are described as a normal clinical feature of plague in connection with the siege of Kaffa from where the Black Death was first shipped to Constantinople and Messina in Sicily and to commercial hubs in mainland Italy where many chroniclers mention buboes [28, 49].

The Black Death broke out in Marseille on 1 November 1348, contaminated by a ship arriving with goods from Genoa. Recently, a paleobiological study of plague graves from the Black Death near Genoa identified *Y. pestis* [49]. From Marseilles, the spread of the Black Death in France can be followed [28, 46]. There are many clinical descriptions of cases of the Black Death which include references to buboes in the same bodily locations, beginning with Guy de Chauliac's clinical description of plague cases in Avignon [50]. From Avignon, the spread of the Black Death can be followed via Lyon to Givry [28] and further to other towns and cities from where there also are extant contemporary clinical descriptions of the disease which include buboes. From Chalon, important (remains of Roman) roads ran north-westwards and northwards in the direction of Reims [28]. In Reims, a list was kept during the Black Death which identified persons who had been miraculously healed from plague disease by intercessory prayer to St. Remi, the city's protector saint. Some of the entries include basic clinical descriptions as proof that the disease really was plague, and many of them refer to buboes with specific locations [51]. All information on normalcy and locations of buboes is consistent with modern bubonic plague, only with bubonic plague and is, thus, a defining feature of bubonic plague [12, 52].

Dubois sums up the two central aspects of the Black Death's spread in France. It is characterized by two features: (1) “almost everywhere, the plague has shown the bubonic form with secondary manifestations”; (2) “usually, it has been stopped or slowed down by the winter and then recrudesced in the spring” [52]. These features are defining features of rat-and-rat-flea-borne bubonic plague [12].

There are other clinical features which could be commented on. Scott and Duncan assert, for instance, that they had proved with the Reed–Frost model that historical plague had a very long incubation period of 32–34 days with a very long period of infectiousness. Purportedly, three weeks of this period was an infectious carrier state, and in addition, Ebola cases are infectious during the 5 days of illness. In all, historical cases of plague disease purportedly lasted 37–39 days of which the diseased were infectious for 26 days [5]. Christakos et al. unconditionally support this information [6]. However, according to the medical study of Ebola Filoviridae disease, infected persons have, as mentioned, a much shorter normal incubation period of 8–10 days [19, 20, 43], there is no contagious carrier state, and contagiousness begins with the course of illness which has a median duration of 8 days (an interquartile range of 7 to 11) [44]. The alleged features of Filoviridae disease asserted by Scott and Duncan and Christakos et al. are illusory reflections of the fallacious use of a SIR model, in this case specifically the Reed–Frost SIR model.

## 6. Results: Conclusions on the Use of the Reed–Frost SIR Model for the Study of Historical Plague and the Invalidation of the Filoviridae Theory of Plague

The narrow mathematical model perspective and the fallacious use of these models are the reasons Christakos at al. do not relate, for instance, to the “complete” presentation of the spread and epidemiology of the Black Death in my 2004 book and do not point out any concrete evidence or analysis that are missing, erroneous, or flawed. They just stay within their own model-based analysis of selected events and conclude the following:

The findings of advanced stochastic modelling and spatiotemporal mapping support the view that Black Death was a different kind of epidemic than bubonic plague.

*No damaging hard evidence has been found against the new proposals concerning the Black Death etiology* [6].

These conclusions which refer to their purported confirmation that the Black Death was a Filoviridae disease are evidently invalid, the outcome of fallacious use of a mathematical epidemiological (SIR) model, and apparent scholarly distance to modern research on bubonic plague and Filoviridae disease. They are incompatible with a full-scale (unselected) presentation of the historical epidemiology of the Black Death [12, 28]. The initial argument of purported findings by advanced stochastic modelling and spatiotemporal mapping with use of Bayesian maximum entropy (BME) has been shown to be at variance also with basic facts and is clearly unsuitable for the present purpose.

This has now also independently been shown by paleobiological studies. By 2014, 20 articles had been published containing, inter alia, 12 studies of biological material obtained in plague graves or pits which with probability or certainty related to the Black Death of 1348–50. The samples have consistently yielded positive identifications of *Y. pestis* in localities as far apart as London and Montpellier. In all, these 20 articles give the outcomes of 45 biomolecular studies of skeletal material obtained in putative plague graves or burial pits in 40 different localities and of hundreds of specimens of individual skeletal remains, 4 of them relating to the Justinianic pandemic and 36 to the second pandemic. In all cases, *Y. pestis* was recovered, not in a single case was there any suggestion of Filoviridae contagion [12, 54].

The historical epidemiological analysis and the paleobiological analysis produce the same factual historical outcome. By invalidating the Filoviridae theory, these forms of analysis also demonstrate that use of (mathematical epidemiological) models to produce proof of aspects of historical reality at some level of tenability is a fallacy of methodology producing illusory reflections of the models. They also show why the proper usage of models is restricted to hypothesis formation.

By use of the Reed–Frost SIR mathematical epidemiological model, Scott and Duncan and Christakos and Olea et al. had allegedly produced strong evidence that the Black Death and historical plague was not bubonic plague caused by transmission of the bacterium *Y. pestis* by rat fleas but was a (variant of) Ebola disease or Marburg disease spread by direct transmission of living contaminated cells from a diseased to a healthy person. Clearly, they were entirely wrong in both respects and for several reasons made clear by Maia in his presentation of the Reed–Frost model and even more by Abbey whose fine contribution is ignored.

## 7. Katharine R. Dean's and Dean Et Al.'s Use of the Reed–Frost SIR Model

### 7.1. Effects of Paleobiological Plague Studies on the Use of Reed–Frost Model or SIR Models

In 2010, the first synthesis of the paleobiological studies of biological material from historical plague graves was presented and in 2016 an updated version [12, 54]. These syntheses showed that paleobiological plague studies had become so numerous, covered such a wide part of Europe, and were so consistent in their identification of *Yersinia pestis*, the plague bacterium, that alternative microbiological theories were excluded. The new scientific discipline had definitely confirmed the results of conventional historical plague studies [28, 54–56]. Nonetheless, a few years later, it transpired that the Reed–Frost model which Christakos et al. had adapted for their use and used to prove that historical plague was a Filoviridae disease, had become an alternative by another name, SIR models, the use of the Reed–Frost model by nominal proxy.

Recently, Dean wrote a thesis for the degree of Master of Science called *Modelling plague transmission in Medieval European cities* [7] at CEES of the University of Oslo. However, not one of the three medieval cities which Dean studied by Christakos et al.'s version of the Reed–Frost model were medieval cities, namely, “Givry in 1348, London in 1563-64, and Florence in 1630-31.” This is due to not recognizing the historical chronological delimitation of the Middle Ages, namely, c. 500–1500 CE, and the population size-dependent definition of a medieval city of at least 10,000 inhabitants, a criterion which the small town of Givry does not satisfy by a huge margin. In the latter case, it is a reflection of the unquestioning accept of Christakos et al.'s unargued and exceptional use of a generalized concept of city.

## 8. Dean's Use of Christakos Et Al.'s Extended Reed–Frost SIR Model to Prove That Plague Epidemics of the Past Were Bubonic Plague Caused by *Yersinia pestis* Transmitted by Human Fleas and Lice

In her thesis, Dean uses Christakos et al.'s work intensively. She acknowledges that the linear relationship or regression that Olea and Christakos found between pre-plague city size and the duration of plague epidemics during the Black Death [30] formed the basis used “to compare the models we made to historical data from the Black Death” [7]. For use of models, material, arguments, and support, Dean refers to the book published by Christakos et al. and the two collateral articles published by Christakos and Olea and vice versa, also in 2005, 25 times in 47 pages of running text, 3 times to the book [6], 21 times to Olea and Christakos' article on the “duration of urban mortality” in the Black Death [30], once to Christakos and Olea's article on “New space-time perspectives” [31]. In addition, she refers once to the 2007 article they published together with Yu, a model-based study on the space-time perspectives on the spread of the Black Death [34]. One should note that the essence of the article on the “duration of urban mortality” in the Black Death and its presentation in the book of Christakos et al. [6, 30] above was shown to be at variance with the basic principle of the function of density in epidemiology and consequently with much empirical data, and evidently was fallacious, another illusory reflection of the model.

These are all works that by intensive use of SIR mathematical epidemiological models in the form of the (extended) Reed–Frost model, according to Christakos et al., allegedly invalidated that historical plague could have been bubonic plague. Instead, it allegedly proved that the Black Death and later plague epidemics of the second pandemic were an entirely different disease, a Filoviridae disease transmitted by direct infection of contaminated cells. Nonetheless, Dean, using the same model and material, allegedly succeeded in reaching an entirely different conclusion, namely, that the Black Death and later plague epidemics of the second pandemic were not a Filoviridae disease spread by direct infection of contaminated living cells but was indeed bubonic plague, caused by the bacterium *Y. pestis* and (purportedly) transmitted by (so-called) human fleas and lice. This shows again that by this use of mathematical epidemiological models seemingly anything can be proved. Because SIR mathematical epidemiological models, such as the Reed–Frost model, can only be used to model viral diseases (according to many strongly restrictive conditions) and because SIR models cannot be used to model “Diseases with multiple hosts, such as insect vectors and animal reservoirs,” Dean's thesis has very serious and basic weaknesses. The pivotal importance for Dean's thesis of the intensive use of Olea and Christakos' article “on duration of urban mortality” [30] shows, for instance, in her statement that it contains a dataset of plague outbreaks in 53 cities that purportedly, without (superfluous) support from a medievalist or historical demographer, had “reliable information for the initial population size and the duration of the epidemic.” Furthermore, the “linear regression obtained in their (Olea and Christakos': my insertion) study formed the basis with which to compare the models we made to historical data from the Black Death.” The lack of historical and demographic competence shows again. Dean's assertion that “weekly and monthly mortality information for historical epidemics can often be obtained from gravestones, burial records, or registered wills” is erroneous from ignorance. Only continuous burial registers for a known population can be used for this purpose and that is not even the case for the burial register of Givry. The appropriate adverb is not often but never.

The recent article by Dean et al. [9], “Human Ectoparasites and the Spread of Plague in Europe during the Second Pandemic,” presents to a large extent the gist of Dean's thesis and the advice given by her supervisors or mentors of whom all but one are among the coauthors. The main difference is that the article contains more examples of SIR models with presentations of mortality data from plague epidemics based on purported correct historical information. However, these examples are presented without (the thorough) source-critical discussion of the historical data that historical demographers would insist upon to ensure the quality and usability of the material and to enable adequate testing of the modelled analogy by relevant empirical data. Unsurprisingly, this material is severely flawed.

For reasons, in this thesis, the works of Christakos and Olea et al. and the use of them are not explicitly mentioned, instead only the term SIR-based models is consistently used, in reality the version of the Reed–Frost SIR-based model used by Christakos et al. This includes also the reuse of Christakos et al.'s model of plague deaths in Givry by month, purportedly with an entirely different outcome: now, it did not show that the Black Death was a Filoviridae disease but was, instead, caused by the bacterium *Y. pestis* and transmitted by human fleas and lice, as in Dean's thesis. This shows again that by this use of mathematical (epidemiological) models, preconceived views can be corroborated, also entirely opposite views, depending on the input and simulation of data. The discussion of Dean's thesis here pertains therefore also to this article but will be supplemented by some direct comments on this article.

## 9. Dean's Use of Christakos et al.'s Reed–Frost SIR Model to Perform Spatial Epidemic Analysis and the Purported Support from W. O. Kermack and A. G. McKendrick's Mumbai Study of Plague

As shown by Dean's extensive use of Olea and Christakos' article on the “duration of urban mortality,” the spatial dimension is not included. Nonetheless, Dean maintains that information on “weekly and monthly” “distribution of deaths is sometimes the only way to understand the dynamics within a past epidemic, without rapid diagnoses and contact tracing.” This statement implies that the conventional identification of epidemic disease by study of the distribution of deaths according to territorial spread, the movement of the disease by diseased according to location of dwelling or street and time, can be dispensed with and substituted by model-based analysis. This statement is supported by an immediately following assertion that “the first SIR-model developed by Kermack and McKendrick was fitted to the number of deaths per week for the 1905-06 plague epidemic in Bombay.” However, Dean uses Christakos et al.'s temporally extended Reed–Frost model with data according to month, not by week. She models, e.g., the burial data of Givry in the same way as Christakos et al., according to month [7]. This temporal choice has the same effect of losing the specific shape of the initial phase of the epidemic which is incompatible with the Filoviridae theory and also incompatible with the human ectoparasite theory but easily compatible with rat-based-flea-borne bubonic plague and as such a defining feature of this modality of plague.

Otherwise, the reference to Kermack and McKendrick's modelled data for the 1905-06 epidemic in Mumbai is superficially correct but, in reality, misleading. The reason is that it is not made clear that Kermack and McKendrick's statistical analysis, clarifying comments, and presentation of the curve of the incidence of deaths by week between 12.17.1905 and 07.21.1906 simply show the number and temporal distribution of deaths. They do not suggest possible use of it for inference to the mechanism(s) of transmission and not for inference to the pattern of intralocal or interlocal spread of the epidemic in an urban centre, in casu Mumbai. They simply assume a priori that, in Mumbai, “plague in man is a reflection of plague in rats” and insert this crucial condition together with other central assumptions. Crucially, they observed nothing in their curve or equations that could suggest otherwise [57], which here is the decisive point when other critical issues are not considered.

Kermack and McKendrick's objective was to gain “insights” into “the process by which epidemics in limited populations run their peculiar courses and end in final extinction,” which is the ordinary use of SIR models. Dean inferred by analogy from Kermack and McKendrick's SIR-based mortality curve to (1) the general usability of similar mortality data by the Reed–Frost version of a SIR-based model to make (2) an allegedly valid inference to mechanism(s) or mode of territorial spread. This is not only a false claim on behalf of the Reed–Frost SIR model but it also ignores that analogies only can be used to construct working hypotheses that can and must be tested by adequate empirical data. Such testing is dispensed with at the cost of making the claim fallacious also on this point. Kermack and McKendrick just assumed that the mortality curve in Mumbai was due to rat-based-flea-borne plague, and they do not suggest that this premise could be inferred from the form of the curve they presented.

Dean's wish to identify the type of epidemic without “contact tracing” refers to the standard procedure of historical demographers when performing such a study. This is the hard work of family reconstitution of a local population on the eve of the epidemic on the basis of parish registers or combined with other registers providing information on households and their location. As the epidemic progressed, this individualized population material permits the identification of the dead (buried) persons and the linking of them to specific households and specific dwellings or houses or streets and, thus, to establish the network or chain of cases according to the spatiotemporal dynamics of transmission of the infection. This has been performed in some way in a substantial number of studies, in relation to, e.g., the plague epidemics in the East Devon town of Colyton in 1645-46, in the northern German town of Uelzen in 1597, in studies of the epidemic in the Derbyshire village of Eyam in 1665-66, in studies of five streets in Bristol in the plague epidemics of 1575 and 1603, and in the district of Jordaan in Amsterdam [47, 48, 58–61]. In the latter study, the patterns of spread of the plague epidemics of 1617 and 1624, according to individualized burials and location of dwellings, were also compared with the pattern of local distribution of deaths caused by the (“Spanish”) influenza epidemic in the same and quite unchanged district in 1918. In this way, the disparate and highly distinct patterns of spread of rat-and-rat-flea-borne plague and spread of flu by interhuman cross infection with virus-infected droplets were demonstrated. Without exception, this very best approach produced conclusive epidemiological evidence that the plague epidemics were rat-and-rat-flea-borne bubonic plague. The presumption or hypothesis that infectious or contagious diseases can be identified by modelled curves showing mortality over time is mainly an illusion. This can only be performed on diseases with a unique pattern of development of mortality over time, which must, then, first be identified and shown to be operative in the type of society in question.

## 10. A Study of a Plague Epidemic by a SEIR Model: The Plague Epidemic in Eyam 1665-66

### 10.1. L. K. Whittle and X. Didelot's Use of a SEIR Model for the Study of the Plague at Eyam

Recently, Whittles and Didelot published a new study of the 1665-66 plague epidemic in the village/township of Eyam using a SEIR mathematical epidemiological model [8]. A SEIR model is a slightly modified SIR model. It mainly differs from the SIR model in the addition of a noninfectious incubation period (latency period), when individuals who are exposed (E) have had contact with an infected person but are not themselves infectious, which represents the addition of the letter E to the acronym. It is supplemented by a Bayesian maximum entropy (BME) mathematical method that was first introduced by Christakos et al. and supported their finding that historical plague was a Filoviridae Ebola or Marburg disease (see above).

Most limitations on the usage of the SIR mathematical epidemiological model and the version of the Reed–Frost model specified above, also the temporally extended version, pertain also to the SEIR model. Use of this variant does not affect the principal methodological problems and the fallacious implications for the epidemiological study of plague epidemics when it is clear, among other things, that plague is a bacterial disease transmitted by hematophagous insects as intermediary agents and with a zootic reservoir of infection. Against this backdrop, it is surprising that Whittles and Didelot can state that their model accounts for the possibility of both rodent-to-human and human-to-human transmission as well as the known household structure (see below). It provides, though, ostensibly, the opportunity to resort to rat-flea-based explanation when the facts on the ground evidently are incompatible with human-to-human transmission by human ectoparasites.

The SEIR model assumes that survivors of a disease carry lifelong immunity [62], which is a standard condition of all basic SIR models. This reflects, as pointed out, that, in practice, the use of (basic) SIR models is limited to viral diseases which often confer lasting immunity in survivors and are spread by interhuman cross infection by physical contact or droplets. The epidemic dies out when “the virus cannot find enough new susceptible people.”

As pointed out earlier, survival of bubonic plague confers only a brief and weak immunity, as is usual with bacterial diseases, and also that historical sources record many cases where a person contracts plague twice and also three times in the same epidemic [11]. This problem is ostensibly resolved because Whittles and Didelot point out that “no recovery is allowed for in their model” [8], which seemingly eliminates immunity as an issue. However, in historical plague epidemics as well as in modern plague in early developing countries roughly around a hundred years ago, the lethality rate was about 80% and the survival rate a significant 20% [20]. This is an additional ground that the model will malfunction. However, also other basic empirical data are neglected to the same effect. They introduce, for instance, a “naïve assumption of a fixed 11-day infection period before death, as employed in previous modelling studies (n. 34-6),” which again also refers to the modelled presumption of no survivors. It also reflects that users of mathematical models generally stay within the arena of mathematical models and hypothetical simulation with input of data. They often show little interest in the huge amount of accumulated medical and historical data on bubonic plague which is needed for adequate empirical testing of the modelled epidemic analogy. They do also not consult any of the outstanding medical standard works on bubonic plague for empirical data, in this case on the duration of incubation and of the course of illness which indicate a usual incubation period of 3–5 days and a course of illness also of 3–5 days, on the average of about 8 days [11].

They also do not recognize that when a rat colony is strongly depleted by plague, the rat fleas which have accumulated on the sharply reduced number of remaining rat hosts, will, at the death of their hosts, swarm into the immediate proximity of a household often without finding new rat hosts. They will after a brief period of starvation leap onto human inhabitants in their proximity and cause multiple quite contemporaneous infections and deaths among the human inhabitants. The assumption that this feature of contemporaneity of household infections, disease, and deaths is a reflection of human-to-human infection and “justifies the use of our model in incorporating human-to-human transmission and household structure” [8, cf. 59] is unwarranted and shows unfamiliarity with primary empirical plague research [11].

As other recent users of S(E)IR models who have had to consider the results of paleobiological studies of plague victims of the past, Whittle and Didelot accept that the epidemic is bubonic plague but also conclude emphatically that their model engenders “conclusive evidence” that interhuman transmission played a predominant role also in the plague epidemic at Eyam. This should according to methodological principles not be possible or be fallacious rather with an analogue model which only can engender hypothetical perspectives or data that must be empirically tested to acquire (some level of) tenability or be invalidated.

Empirical testing should include the prevalence and levels of bacteraemia in human plague cases which would determine the possible prevalence and level of infection of feeding human fleas or lice and whether or not feeds would meet the criteria for transmission of potentially lethal doses for human beings. Whittles and Didelot do not really relate to the question of human plague bacteraemia. As for the necessary condition of a viable mechanism of transmission, they argue for the hypothesis of early-phase transmission [8]. As mentioned above, this hypothesis has been invalidated for human plague and may be significant only in highly susceptible rodent populations experiencing a high flea burden [3, 4, 50]. It will also be briefly explained below.

### 10.2. Sources and Household Reconstitution of the Community of Eyam

Whittles and Didelot claim that they combined the 1664 hearth tax record for Eyam and the Eyam parish register to reconstruct the household structure for all persons living in the parish during the time of the plague. This implies that their analysis purportedly also includes the historical demographic method of family/household reconstitution which permits to trace the dead to their habitation as they are entered in the parish burial register with usable information on location of dwelling. This should allow a good spatiotemporal analysis of the epidemic, following in considerable individual detail the territorial spread of the epidemic in the township over time by individual deaths. Importantly, this should make the use of a mathematical epidemiological model quite superfluous because it represents the empirical testing of the modelled analogy that otherwise would, on methodological principle, be required for empirically valid epidemiological inference. There is, however, scant evidence of such material and demographic epidemiological analysis in the article.

Previous studies of the plague epidemic in Eyam are entered in References, but Whittles and Didelot do not mention that, over a generation ago, Bradley studied the plague epidemic in Eyam on the basis of a professional and complete family/household reconstitution combining the use of the parish registers and the hearth tax register [59]. It is also not mentioned that Bradley's reconstitution was used for a stringent in-depth epidemiological analysis by Coleman, the epidemiologist, also over a generation ago. Both scholars emphatically assert that the epidemiological analysis shows the characteristic features of rat-based-rat-flea-borne bubonic plague [64].

### 10.3. Demographic Analysis of the Epidemic in Eyam

At the time, dead were normally buried the next day, which puts the information in the burial register in temporal perspective. The first plague victim was interred on 7 September after 3 days of illness and would then have been infected on about 30 August, and the second plague burial occurred on 22 September, 15 days later. This period is much too long for interhuman infection by human ectoparasites according to the known duration of the period of incubation and illness. It implies a sudden end of the spread of the incipient epidemic, which did not occur and, thus, invalidates the human ectoparasite assumption. Instead, the period of delay fits nicely with rat-and-rat-flea-borne plague where the flea causing the first infection or another flea from the same source would have sought out its true host and triggered a rat epizootic. The rat epizootic would take 10–14 days before the colony was so depleted that rat fleas that had accumulated on the remaining rats, at the death of their host often would not find a new host. After 3 days of starving, these fleas would feed on human beings in their proximity, and the first death would usually follow about 2 × 3–5 days later. This is a process that would take in all 19–27 days, on the average 23 days [12], which in this case would be on 22 September, when the second death occurred. As pointed out earlier in the discussion of the Black Death epidemic in Givry, the 23 days elapsing between the first plague case and the second represent proof of the initial phase of an epidemic of rat-and-rat-flea-borne bubonic plague. The Indian Plague Research Commission (IPRC) early established that there was an interval of 3.5 weeks [54].

Whittles and Didelot point out that previous use of SIR models on this epidemic only related to the second half of the epidemic because it could not explain its first phase [8]. This shows again how problematic the use of SIR models are in this field of research and contains a serious warning against use unless the dysfunction is positively explained, which is instead passed over. This statement also includes the sharp decline in burials from October to (almost) insignificant superincidence compared to the normal number of burials in these months, until the epidemic recrudesced with a vengeance in June. The sharp decline from the end of October and its duration through the winter months and the early spring months are incompatible with notions of interhuman transmission of plague because it includes too long intervals without spread of infection and new victims. There is, e.g., no plague burial between 1 and 15 and 28 of January [59, 65].

This decline and (near) disappearance of the epidemic are also not compatible with louse-borne plague which Whittles and Didelot, like Dean and Dean et al., include as important agents of plague transmission [8]. Wintry weather should strengthen human-to-human cross infection by lice because people crowded closer together in habitations and also in beds at nights, with warmer/thicker and less washed beddings to keep warm and to restrict fuel expenses; and during the day, they wore more and less washed and increasingly louse-infested clothing. These are the main reasons louse-borne rickettsial epidemic diseases, such as exanthematic typhus and quintan fever (trench fever), display strong powers of spread in the winter months [54, 66, 67], which should be replicated by plague, also in Eyam, if the epidemiological premises were tenable, which they evidently are not.

The steep fall in plague cases with the advent of cold weather should acutely raise the questions of whether human plague cases actually functioned as sources of potential lethal doses of infection of feeding human lice and fleas. All advocates of the human ectoparasite theory of plague transmission have in common that they avoid raising this crucial question and neglect to obtain the relevant empirical data on human bacteraemia (see below). In addition, there is the likewise crucial question of the ability of human fleas and lice to transmit the infection, their vector capacity, which is another neglected but necessary condition for the viability of the hypothesis of a predominant (or significant) role of human ectoparasites in the transmission of plague.

It is not correct that this seasonal pattern of a plague epidemic, the sharp decline with cold weather followed by recrudescence with the advent of warmer spring weather, could be a unique case [8]. This is the typical feature of plague epidemics that break out in the autumn and do not have time to complete the course of spread among the local rat colonies before halted or sharply reduced by the advent of cold autumnal and wintry weather. Plague epidemics with this seasonal pattern are called transseasonal epidemics [12]. This argument is evidence of deficient knowledge of historical plague epidemics.

As fur fleas, rat fleas enjoy a comfortable microclimate in the rat fur also in wintry climate and with ready opportunity for feeding. The rat-flea population declines because the replenishment is hampered by chilly floors or ground with harmful effects on ova and larvae. The crucial point in the decline is not the slow decline in the flea population, as assumed by Whittles and Didelot, but that the prevalence and levels of plague bacteraemia in rats sharply decline when temperatures fall below about 10°C. For this reason, the rates and levels of plague infection acquired by feeding rat fleas fall steeply, and the epidemic returns to a smouldering enzootic form until the advent of warmer spring weather (if it does not become extinguished) [26, 68, 69]. For this reason, the transseasonality of bubonic plague is also a unique and defining feature of rat-based-rat-flea-borne bubonic plague [12].

## 11. The Functions of Human Plague Cases as Sources of Infection of Feeding Fleas and Lice: Mathematically Modelled Epidemic Hypotheses Seen in the Empirical Light of Human Plague Bacteraemia and Purported Early-Phase Transmission

### 11.1. Empirical Testing of the Claim That SI(E)R Models Support That Human Fleas and Lice Function as Crucial Vectors of Historical Plague Epidemics

In recent years, several scientists have claimed that human fleas and lice were the predominant vectors of the plague epidemics of the past, for instance, Dean, Whittles and Didelot, and Dean et al., all using Reed–Frost or other close variants of S(E)IR mathematical epidemiological models which do not permit study of epidemic disease transmitted by insects. Although on the methodological principle, analogue models cannot produce evidence with empirical status, these scholars refer to the outcome of their use of S(E)IR models as evidence [7–9]. Dean and Dean et al. also refer as evidence to the fact that human lice and human fleas have been found to be infected by plague bacteria after having fed on human plague patients with bacteraemia, i.e., with plague bacteria in the blood stream. Evidently, this does not per se contain indication of vector capacity or ingestion of lethal doses. They refer to G. Blanc and M. Baltazard's two-page 1942 poster inspired by a tiny endemic rural outbreak in Morocco (90 cases) in 1941 [70], without mentioning that it was immediately severely criticized in a long article by Girard [71], a former leader of the French research effort on plague in Madagascar in the capacity of director of the Institut Pasteur de Anatanrivo 1922–1940 and then director of Institut Pasteur de Paris, which must have been a serious matter for two scientists at Institut Pasteur du Maroc. They do not refer to Blanc and Baltazard's 1945 treatise [72] that has been analysed by several scholars, consistently highly critically as methodologically and materially severely flawed, a discussion that have been gathered together and presented in a form suitable for consideration and discussion [33, 54, 73]. All blood sucking insects including mosquitoes, bed bugs, and ticks and also flies, ants, beetles, and cockroaches which have ingested bloody cough from pneumonic plague patients or other plague material become infected but no one has argued that they function as (significant) vectors of plague contagion [11, 40]. The fact that insects feeding on bacteraemic human blood become infected does not imply and cannot serve as evidence of a vectorial capacity for plague disease or transmission of lethal doses.

### 11.2. Are Human Fleas Vectors of Plague? Human Plague Bacteraemia and the Size of Blood Meals Taken by Human Fleas Seen in relation to LD

A significant or central vectorial role in plague epidemics for human fleas and lice are dependent on several necessary conditions that generally are not considered by proponents of human ectoparasite hypotheses of plague transmission: (1) the prevalence and levels of human plague bacteraemia; (2) the general size of blood meals ingested by human fleas and lice; (3) the consequent number of ingested plague bacteria; (4) the lethal dose (LD) of plague bacteria for human beings, normally expressed as the number causing death for half of those infected, LD_50_; and (5) efficient mechanism of transmission by lice and by fleas. The factual answers to all these crucial questions can be ascertained. The problems and perspectives involved are too comprehensive to be presented and discussed in detail here, but the gist can be presented also briefly and satisfactorily filling the lacunae on these matters in the discussion.

Mass studies of human plague bacteraemia were performed on patients in Mumbai's plague hospitals around 1900 and several quite large studies by American physicians during the Vietnam War when there were quite big plague epidemics with 4,500–5,600 recorded cases in the three peak years of 1965–67 [54, 74, 75]. These studies showed that, respectively, not more than 45% and 30% of human plague cases developed plague bacteraemia, or conversely that 70–55% did not and could not serve as sources of infection for feeding fleas or lice. 244 individual studies of the level of bacteraemia were also performed at some advanced point of the disease or repeatedly during the (remaining) course of illness which supplemented the 28 cases individually studied by IPRC [76], in all 272. These studies showed that the huge majority of bacteraemic plague patients developed only slight levels of bacteraemia and that levels did not change significantly during the last half or latter course of illness and also that there was an insignificant incidence of change from nonbacteraemic to bacteraemic form [54].

The IPRC's study of the fully distended midgut of a female black-rat flea showed a feeding capacity of 0.5 *μ*L. Recently, a new study showed that the average blood meal taken by the black-rat flea *X. cheopis* was 0.41 *μ*L for females and 0.18 *μ*L for males [54, 77]. These studies may not differ, and it is the size of blood meals that is the crucial measurement. A preliminary study of blood meals taken by female human fleas (*Pulex irritans*) showed an average size of 0.32 *μ*L [78]. Because the latter study was considered preliminary, the measurement of the average size of female rat fleas' meals will be used below to ensure that estimates are based on solid empirical data, are within wide margins of safety, and error on the high side.

These data show that female or male rat fleas must ingest blood containing on the average ∼2440 or ∼5560 plague bacteria/mL, respectively, in order to become infected by 1 plague bacterium, which represents the infection-level divisors or gauges. According to the 272 individual measurements of human septicaemia, at least 90% of all plague patients will not infect a feeding female flea, which takes by far the largest blood meals, with a single plague bacterium during the course of plague disease [54].

When fleas ingest sufficiently contaminated blood, bacteria follow into the stomach, technically called the ventriculus or midgut. In addition to the midgut, the stomach system of fleas also consists of a proventriculus or foregut with a valve function allowing fleas to make, relatively speaking, huge intakes of blood because the valve prevents the blood in the strongly distended midgut after a feed from forcing its way back out. After a blood meal, fleas do, therefore, rarely attempt to feed more frequently than once in 24 to 72 hours, which represents the minimum interval between feedings, normally “perhaps every 4 or 5 days” [79, 80]. In the meantime, fleas digest the blood meal and pass it gradually into and through the lower digestive tract (hindgut). Only when most of the meal is digested, will fleas again feel hungry and start the search for a new feeding opportunity. This means that the digestive process takes on the character of self-purification of the infection so that, as a preliminary consideration, at least 97% of feeding fleas which have fed on human plague diseased will for all practical purposes be infection free and noninfectious at the time of the next blood meal taken from plague-diseased human beings.

This line of reasoning can be developed further. Among the 272 plague patients who were individually examined for (level of) bacteraemia, there were 7 cases with the relatively high levels of 10^6^–1.5 × 10^6^ bacteria/mL of blood. They can be designated statistical outliers because there is a wide gap down to the next level of highest recorded levels of bacteraemia. By very far, the highest level of human plague bacteraemia on record is a unique or extreme outlier with 4 × 10^7^ bacteria/mL [54]. These 8 statistical outliers, who constitute 2.9% of all individually examined human plague cases for (level of) bacteraemia, probably represent the normal prevalence of cases with primary bacteraemic plague. Fleas feeding on the 7 human cases with a level of bacteraemia of 1–1.5 × 10^6^ will, according to the sex-specific infection gauges, ingest ≥180/410 *Y. pestis* bacteria. Taking into account fleas' ability of self-purification, it seems likely that also these cases will, for all practical purposes, be infection-free at the time of the next blood meal.

Only the extreme outlier with a level of bacteraemia of 4 × 10^7^ bacteria/mL, which, according to the sexed infection gauges, would infect feeding female or male fleas (*X. cheopis*) with, respectively, ∼16,400/∼7,200 bacteria, seems, in this context, to be of interest with respect to potential for infecting feeding fleas with LDs. This technical and statistical argument, based on hard scientific data, implies that 99.6% of all fleas which feed on human plague cases for all practical purposes will be infection free at the time of the next blood meal.

This is concordant with the fact that transmission of plague by human fleas is not observed in large-scale laboratory experiments [81]. IPRC noted that *Pulex irritans* could transmit plague to healthy guinea pigs if fed on extremely bacteraemic rat blood, 10^9^ bacteria/mL, which never occurs in human beings by vast margins. They recorded 3 successful transmissions out of 38 experiments with batches of 20 fleas, i.e., 720 fleas, a transmission rate of 0.14% under extremely favourable conditions. Because transmission by blockage/biofilm was not known, the mechanism of transmission of lethal doses of plague bacteria to the guinea pigs was not observed and will remain uncertain [82]. Guinea pigs are, however, exceptionally susceptible to plague infection, studies show that infection by 1 plague bacterium killed two-thirds of guinea pigs, i.e., LD_63_ = 1 [10]. This means that the transmission of lethal doses of plague infection to these guinea pigs probably occurred by the mechanical method, biting by mouth parts still soiled with blood containing one or a few plague bacteria, which would be without significance for human beings. This observation is concordant with the fact that transmission of bubonic plague by human ectoparasites never has been observed during bubonic plague epidemics.

Plague experiments cannot be performed on human beings for evident ethical reasons, and rodent data must suffice as a base for a tentative inference to LD_50_. Relevant data are few also in this case. 90% of black rats survived primary inoculation with 5,550 plague bacteria, corresponding to LD_10_, LD_50_ must be significantly higher. LD_50_ of a species of highly susceptible ground squirrels was 6,070 plague bacteria [54], which arguably could be usable as a cautious LD_50_ for human beings of ∼6,000 *Y. pestis* [54]. This means that female/male human fleas must feed on blood containing at least 2.7 × 10^6^ or 7.2 × 10^7^ bacteria/mL to ingest this number of plague bacteria. By a huge margin, this requirement is met only by the human case with the unique/extreme level of bacteraemia of 4 × 10^6^ or 0.4% of all 272 human plague cases individually examined for level of plague bacteraemia. This evidence also explains that no concrete case of interhuman cross infection of bubonic plague was observed in the big plague epidemics in India, China, Java, and Madagascar or in the large epidemics in Egypt and Vietnam, according to the huge syntheses of plague studies given in the standard works on plague. This is put in perspective by the fact that native huts and hovels swarmed with human ectoparasites [54].

### 11.3. Are Human Lice Vectors of Epidemic Plague? Human Plague Bacteraemia and the Size of Blood Meals Taken by Lice Seen in relation to LD

Some experiments with lice are, as mentioned, used to advocate an important vectorial role for this ectoparasite in plague epidemics. Dean and Dean et al. refer for evidence to the fact that human lice have also been found to be infected by plague bacteria after having fed on human plague patients with bacteraemia. However, as pointed out, this does not constitute evidence of ingestion of LDs of plague bacteria or of subsequent development of LDs in the gut or the hindgut before the next feed. It is evidently also not evidence of capacity of transmission, for instance, by the rubbing of infected faeces into itching bite wounds by the hosts themselves, according to the rickettsial mode of infection. Dean and Dean et al. refer only to Blanc and Baltazard's two-page 1942 poster inspired by a tiny endemic rural outbreak in Morocco in 1941 [70], without mentioning that it was immediately severely criticized in a long article by Girard [71] as shown above. They do not refer to Blanc and Baltazard's 1945 treatise [72], which, as shown above, has been analysed by several scholars, consistently highly critically.

Blanc and Baltazard performed experiments with 63 lice which presumably were predominantly adults, an assumption that will also maximize the volume of blood and possible contamination by *Y. pestis*. Because there tend to be more females than males in a normal lice population [83], it seems reasonable to suppose that 60% were females. Recent research has shown that an adult female louse (imago) imbibes, on average, 0.0001579 mL = 0.1579 *μ*L per blood meal, an adult male 0.0000657 *μ*L [84], and take on the average three feeds per day [79, 84, 85]. This means that 63 adult lice, distributed on 38 females and 25 males on the average, imbibe 0.0229281 mL = ∼0.023 mL or ∼23 *μ*L of blood a day. For practical or pedagogical reasons, the following estimates will take as a point of departure that a level of bacteraemia of 1000 *Y. pestis*/mL of blood implies that each *μ*L of blood contains on the average 1 plague bacterium. This implies that a batch of lice with the presumed composition and feeding habits which had ingested 23 *μ*L of blood in the past 24 hours would altogether be infected by 23 plague bacteria when feeding on a person with a level of bacteraemia of 1000 bacteria/mL of blood, a level measured only in one of 271 individually studied human plague cases, albeit with large margins. Also as an approximate estimate to be considered within substantial margins of error, this is an important analytical tool which, in the light of the presented data on human plague bacteraemia, puts in perspective claims that lice can play an important part in the transmission of bubonic plague, a negligible part would be more accurate, as maintained by Girard in 1943 [71].

Blanc and Baltazard neglected to examine the group of 90 plague cases constituting their research material with respect to prevalence and levels of human bacteraemia and thus their possible function as sources of infection of feeding ectoparasites. However, quite accidentally, as it seems, they withdrew blood from 2 of the 90 plague cases (nos. 4 and 24), who were examined by haemoculture. It transpired that that one was bacteraemic, i.e., 50% [72]. This shows that they had the technical prerequisites for studying the prevalence of bacteraemia and also, quite likely, levels of bacteraemia, also during the course of illness, such as the IPRC had performed in India over a generation earlier [76]. Blanc and Baltazard do refer to their laboratory and the local lazaretto [72]. Evidently, they had the opportunity of testing the basic and crucial premises of their hypothesis that human plague cases quite generally develop bacteraemia and at sufficient levels to infect feeding human fleas and lice with sufficient doses for epidemic lethal transmission but refrained from using it. They also could have availed themselves of IPRC's data.

According to the feeding capacity of female and male human lice, they must ingest blood with levels of bacteraemia of, respectively, ∼6,300 and ∼15,200 *Y. pestis*/mL in order to take in 1 plague bacterium, which is the infection gauge or infection-level divisor of female and male lice, respectively. This means that 3 of the 272 human plague cases examined for level of bacteraemia would infect feeding human female lice, on average, with nearly 2 bacteria per blood meal, 5 cases with inaccurate measurements would infect the feeding female lice with 0–>∼160 plague bacteria, 7 cases with ∼160–240 bacteria, and only the extreme statistical outlier with 4 × 10^7^ would be a source of a presumed LD for human beings, and only this case would infect a male louse and then with 66 bacteria. These data show that lice, for all practical purposes, will not ingest lethal doses of plague by feeding on human plague cases and by vast margins. Because lice feed 3 times/day and with simultaneous profuse defecation, there will not under any circumstances be time for significant bacterial growth in the gut or hindgut.

Plague bacteria are not evolutionarily adapted to preserve virulence during the passage through the hindgut. Defecated plague bacteria have strongly reduced virulence. Also, “the faeces do not, as a rule, contain many bacilli and soon dry up” [86, 87]. Large-scale experiments with fleas have consistently shown negative or highly restricted results with respect to possible transmission of plague bacteria by rubbing faeces into bite wounds or scarified skin on guinea pigs which are extremely susceptible to plague infection, according to the lice-typhus model [80, 85, 87], studies which are not mentioned. In nature, this potential route of infection would be strongly negatively affected by the fact that “fleas seldom deposit faeces when feeding and the reactions following the bites of rodent fleas rarely cause itching” [80, 85].

*Y. pestis* has also not adapted by highly reduced size to being transmitted by being scratched into bite wounds but preserved a stable and normal size [54]. Plague bacteria are evolutionarily pro-selected according to high virulence which is crucial for the ability to produce biofilm or blockage and consequent transmission (pigmented hemin storage, hms+), while low-virulent strains are out-selected [88, 89]. This process of evolutionary selection accounts for the very high and stable virulence and also size of plague bacteria [33]. This stands out in contrast to *Rickettsia prowazekii* that by evolutionary adaptation to be scratched through tiny bite wounds has only one-sixth the size of plague bacteria [54]. This is the reason that human plague cases normally develop buboes because the contagion is deposited by fleas at an intradermal level in the catchment area of the lymphatic system and is drained to lymph nodes which swell upon infection. Only human plague cases who develop secondary bacteraemic plague by leakage of bacteria into the blood stream from buboes worn down by toxins also display petechia. In contrast, cases of exanthematic typhus rarely develop buboes while petechia are a regular feature, confirming that the bites of lice and the size of *Rickettsia prowazekii* produce regular bacteraemic infection by scratching of lice faeces or the body juices of crushed lice through the bite wound.

Only Blanc and Baltazard's poster with considerations on experience with a few rural plague cases in Morocco in 1941 is mentioned in the discussion of the possible role of human lice in plague transmission. The negative results of fine studies on the possible vectorial role of lice by French researchers in Madagascar are not referred to [27, 90].

In addition, advocates of an important vectorial role in plague epidemics for human lice also refer to a more recent article presenting laboratory experiments. Experiments with lice performed by Houhamdi et al. allegedly support the notion of a vectorial role for human lice [91]. Nonetheless, arguably they are highly unsatisfactory in many respects [54]. In these experiments, lice were fed on rabbits inoculated with 10^9^ cfu (colony-forming units) of plague bacteria without relating to the questions whether transmission of such a huge load of plague bacteria occurs in nature, the usual size of infections ingested by lice on naturally plague-infected rabbits, and the relative incidence of possible consequent transmission of plague by lice. Another matter is the relevance of the choice of rabbits in relation to epidemic potential in human habitation and social contexts. They also do not consider whether collective movement of groups of infected lice would occur in nature. Lice are evolutionarily adapted to stay in a fixed position on the host and have innately very poor ability to move (by crawling) and were, in this case, helpfully transferred by scientists simultaneously onto other animals. The experiments are also performed without a real consideration of the experiments' relationship with human plague bacteraemia which would be the source of infection in plague epidemics, only in a highly selective and unrepresentative manner. These experiments were basically similar to the experiments purportedly proving the efficiency of early-phase transmission that Hinnebusch pointed out were based on “important parameters with little or no experimental [i.e. empirical] support” [2].

In its first set of experiments, IPRC performed also an experiment where plague-diseased guinea pigs and healthy guinea pigs were allowed to run free in a room, simulating the situation in an infected rat colony. When the fleas were removed but not the lice, nothing happened, despite the fact that the LD_63_ of guinea pigs is 1 plague bacterium; when fleas were introduced, the epizootic, once started, spread “from animal to animal, the rate of progress being in direct proportion to the number of fleas present,” which settled the matter [92]. The IPRC also studied the prevalence and level of plague bacteraemia in black rats [93], data which also could usefully have been applied in this context because human beings live much more often in proximity with commensal or peridomestic black rats than with laboratory rabbits. Houhamdi et al.'s experiments can mainly be considered artificial laboratory constructions without relevance for the understanding of epidemic plague.

Houhamdi et al. used bacterial strains of the plague “biovar Orientalis” [91], which has been shown not be representative of historical plague. This means, as pointed out, that coauthors M. Drancourt and D. Raoult's consistent identification of this “biovar” in their historical research must be due to laboratory contamination [94]. As such, this is a sufficient condition for invalidation of the paper.

The prevalence and levels of plague bacteraemia among black rats are hugely different from human plague bacteraemia: rats (and guinea pigs) generally develop nearly 1000 times higher levels of bacteraemia than human beings, that is, as a comparison between cases who develop bacteraemia and are comparable, which only a minority of human plague cases does. In reality, therefore, the real difference is much bigger [54]. Plague-diseased black rats are hugely superior sources for infection of feeding fleas and lice in comparison with human plague cases. A large proportion of plague-diseased rats will infect feeding fleas with numbers of plague bacteria conducive to formation of blockage/biofilm [93], which is a necessary condition for transmission of LDs, also to human beings, as also emphasized in several research articles by Hinnebusch and with various teams of coauthors in recent years [2–4, 88, 89, 95, 96].

These basic epidemiological and bacteriological data speak clearly and loudly for themselves and suffice for invalidation of the hypothesis of a predominant or important role for human fleas and/or lice in the transmission of bubonic plague, also for a tiny role. There are also other reasons representing independent sufficient conditions for invalidation of any version of the hypotheses of a role for human fleas or lice in the epidemiology of plague [54]. This explains that not any of the authors of the outstanding standard works on plague, who also have in common a life-long combat of big plague epidemics in various early developing countries, in the field and in laboratories, allotted any significant role to human ectoparasites, although the huts and hovels of the natives were heavily infested.

## 12. Comments on the Invalidated Hypothesis of (Epidemic) Early-Phase Transmission

Dean, Whittles and Didelot, and Dean et al. all resort to the so-called early-phase transmission, a form of transmission without blockage, as the means of transmission by human ectoparasites [7–9]. They do not attempt to explain how hypothesized lethal doses of plague bacteria from a preceding feed should be transmitted against the forceful stream of a new feed back into the next bite wound, a formidably counterintuitive proposition. As pointed out earlier, the hypothesis of early-phase transmission has been invalidated as a significant factor in plague epidemics. Taken together, this leaves them without substantiating evidence on the presumption on human plague bacteraemia as sufficient source of lethal bacterial doses and without a functional means of transmission of plague infection. This means not only that the analysed use of S(E)IR models is highly problematic to put it cautiously but it is also without empirical corroboration of crucial conditions, in technical methodological parlance, crucial assumptions are arbitrary.

When Whittles and Didelot assert that “the model suggests” that 73.0% of infections in Eyam came from human-to-human transmission (95% credibility interval: [67.3%, 78.2%], with the remaining 27.0% of infections caused by rodents (95% credibility interval: [21.8%, 32.7%]), these assertions are, evidently, illusory reflections of the models. This is also the case with the use of models to underpin assertions with purported empirical status to the effect that human ectoparasites could play an important role in the plague epidemics of the past. Dean states, e.g., that “With this model of louse-borne transmission we can now show that this mode produces a pattern of transmission in towns and cities that is similar to those from the Black Death” [7]. The assertion that “Mathematical modelling can provide strong insight into mechanisms of plague transmission for past epidemics,” which indicates a clear empirical status for the insight, is also erroneous. Mathematical epidemiological models are analogues that can only be helpful for generating and developing hypotheses suitable for empirical testing. Testing has been performed above and invalidated the model-based results.

On the topic of models suitable for identification of an epidemic disease and the means or mechanism of transmission, I believe, there is only one viable mathematical epidemiological model which has produced valuable and tenable results. It was specifically developed by Roger Scofield, one of the previous century's leading historical demographers, to test the epidemiological and microbiological character of the plague epidemic in Colyton (Devon, England) in 1645-46 on the basis of professional application of the demographic technique of family reconstitution of local society. This allowed him on a strong empirical basis to uncover the real spatial structure of epidemic spread and its impact on various types/sizes of households. He concluded on convincing grounds that it displayed the characteristic features of rat-based-rat-flea-borne bubonic plague [54, 58].

## 13. Conclusions: The Uses and Limits of Mathematical Epidemiological Models in Historical Plague Research

In recent years, several teams of scientists have used mathematical epidemiological SIR or the closely related SEIR models to determine the microbial agent and the dynamics of spread of the Black Death and subsequent plague epidemics of the Second Plague Pandemic (1346-c. 1690). Using basically the same models, they have, nonetheless, identified two entirely different diseases, also different from the rat-and-rat-flea-borne bubonic plague presented in the standard works on plague: historical plague (1) was a Filoviridae disease (e.g., Ebola disease); (2) a bacterial disease caused by the bacterium *Yersinia pestis* transmitted by inter-human cross infection by human fleas and lice and, (3) in the case of human fleas, was transmitted by early-phase transmission (EPT).

In the methodological principle, if basically the same model can be used to “prove” or corroborate incompatible theories, in casu that historical plague was two entirely different diseases, something must be fundamentally wrong with the model or, alternatively, the model is used erroneously, outside the range of application. Here, this problem is discussed for the first time.

In the Introduction, it is pointed out that, methodologically, models are inherently analogues. As such, models can be used to engender working hypotheses by the presumed significance of seeming similarity. The essence of models is that they provide guidance for the construction of working hypotheses that can be empirically tested or for experiments which can provide empirical data suitable for testing. Generation of working hypotheses is highly important and valuable in all forms of research and attests to the scholarly value of models but working hypotheses must be empirically tested to produce evidence on some aspect of social reality at some level of tenability.

The conclusion is that these incompatible outcomes (and the support for early-phase transmission, EPT) are due to erroneous use of mathematical S(E)IR epidemiological models in two crucial respects. (1) The use does not take into account the range of applications and the limitations pointed out by Theodorson and Theodorson due to these models' basic methodological character as analogues. (2) The incompatible outcomes are also due to the ignoring of the limitations in the uses of these models emphasized by Maia and Abbey. Centrally, they argue that the uses of these models are restricted to viral diseases which give survivors lasting immunity and that are transmitted by direct cross infection and not by intermediary agents such as insects. This means that these models will malfunction if the studied historical plague epidemics were bacterial, e.g., caused by *Y. pestis*, and/or had multiple hosts, e.g., were transmitted by insects such as human fleas and lice or were rat-and-rat-flea-borne according to the medical and historical standard works on plague.

The outcomes of the use of these models with respect to historical plague epidemics can be empirically tested: (1) Paleobiological studies consistently show that historical plague was caused by the conventional plague bacterium *Y. pestis* [54, Ch. 1.5; 12, Ch. 10]. (2) Studies of the prevalence and levels of bacteraemia in human plague cases show that they cannot serve as sources of infection of feeding human ectoparasites that can enable them to transmit lethal doses of plague except in exceedingly rare cases, irrespective of the hypothesis of early-phase transmission (ETP). (3) Studies show that human fleas normally hardly have vector capacity for plague infection and potentially can transmit plague only exceedingly rarely. (4) The proposed role of ETP has been invalidated as a possible or potential epidemic mechanism of epidemic transmission by Hinnebusch et al. [3, 4].

These data represent conclusive empirical evidence which can explain that all scholarly studies of historical plague epidemics by use of mathematical epidemiological S(E)IR models have produced erroneous outcomes by malfunction because they have been used outside their range of application. This evidence explains why the S(E)IR models malfunction when they are used to identify the microbial agent and epidemiological dynamics of the plague epidemics of the past and indicate completely different diseases and mechanisms of transmission. (5) Studies show that plague-infected black rats have a prevalence and levels of plague bacteraemia that make them extremely good sources for heavy infection of fleas that can enable them to transmit lethal doses of plague bacteria. (6) Laboratory studies show that the black-rat flea *Xenopsylla cheopis* has a superior vector capacity for transmission of lethal doses of plague.

(7) The historical study of plague epidemics, for instance, the complete history of the Black Death (1346–1353) or the epidemics of the Second Plague Pandemic (1346-c. 1690), shows that they consistently display the characteristic patterns of seasonality and spread of rat-and-rat-flea-borne plague of *Y. pestis* [12, 28, 54]. This is in complete concordance with the standard works on plague and also the historical views provided by diachronic historical comparison by the authors [11, 26, 41].

Mathematical epidemiological S(E)IR models can be useful and valuable in the study of epidemic diseases: (1) These models are useful for generation of working hypotheses which is crucially important in all forms of research. (2) S(E)IR models are also useful in the study of a wide range of viral diseases which provide survivors lasting immunity and are transmitted by direct cross infection. The empirical testing of such models in this article shows why they malfunction in historical plague studies and confirmed the limitations in the range of application indicated by Theordorson and Theodorson and by Maia and Abbey.

The use of mathematical S(E)IR models in the study of historical plague epidemics is also analysed on the basis of the historian's craft and knowledge of medieval and Early-Modern society and contemporary sources. The analysis of the scientists' input of historical data in the models shows deficient knowledge of historical society and sources which contribute to the malfunctioning of the models. This point suggests the probable usefulness of interdisciplinary cooperation and that historians can make valuable contributions in historical studies performed by scientists.
